# Molecular diagnosis of inherited platelet disorder via a targeted whole-exome virtual gene panel: a 5-year institutional experience

**DOI:** 10.1016/j.rpth.2026.103413

**Published:** 2026-03-12

**Authors:** Wenying Zhang, Kevin E. Todd, D. Brian Dawson, Lori Luchtman-Jones, Joseph S. Palumbo

**Affiliations:** 1 Division of Human Genetics, Cincinnati Children’s Hospital Medical Center, Cincinnati, OH, USA; 2 Department of Pediatrics, Cincinnati Children’s Hospital Medical Center and University of Cincinnati College of Medicine, Cincinnati, OH, USA; 3 Division of Hematology, Cincinnati Children’s Hospital Medical Center, Cincinnati, OH, USA

**Keywords:** blood platelet disorders, thrombocytopenia, hematologic neoplasms, high-throughput nucleotide sequencing, genetics

## Abstract

**Background:**

Inherited platelet disorders represent a prevalent but inadequately understood subset of bleeding diatheses, often with elusive causative genetics. The low rate of molecular diagnoses highlights the critical need for advanced genetic tools to enhance risk stratification and therapeutic personalization.

**Objectives:**

To address this, we retrospectively reviewed our institutional experience using a whole exome sequencing–based platelet virtual gene panel in patients with suspected inherited platelet disorders to determine the frequency of variant identification, rate of diagnosis assignment, and clinical phenotype associated with variants in the 67 evaluated genes.

**Methods:**

Testing was performed clinically on 409 patients with genetically undefined platelet function abnormality and/or thrombocytopenia. Results of each gene panel underwent expert review by faculty in human genetics and hematology.

**Results:**

Of the 409 patients analyzed, 59 (14%) had variants that were deemed diagnostic for their clinical phenotypes: 37 associated primarily with thrombocytopenia, 4 with platelet function defects, and 18 impacting both platelet number and function. Notably, 22 diagnostic variants were identified in genes associated with familial myelodysplastic syndrome or leukemia, underscoring the far-reaching clinical implications of this testing.

**Conclusions:**

The whole exome sequencing–based virtual panel approach thus provides molecular diagnoses that would otherwise remain elusive, offering patients and families both a diagnostic clarity and crucial information for genetic counseling and medical management. Moreover, the identification of variants associated with hematologic malignancies expands the scope of implications of risk stratification beyond that of platelet pathology, to include informed decision making about surveillance for malignant transformation.

## Essentials

• Inherited platelet disorders lack adequate genetic explanations.
• We tested 409 platelet disorder cases via a targeted whole exome sequencing–based virtual gene panel.
• Genetic causes were found in 14% of cases, and 22 cases had variants associated with cancer risk.
• The findings clarify the diagnosis, reveal risk to leukemia, and guide care and family counseling.

## Graphical abstract

**Figure:**
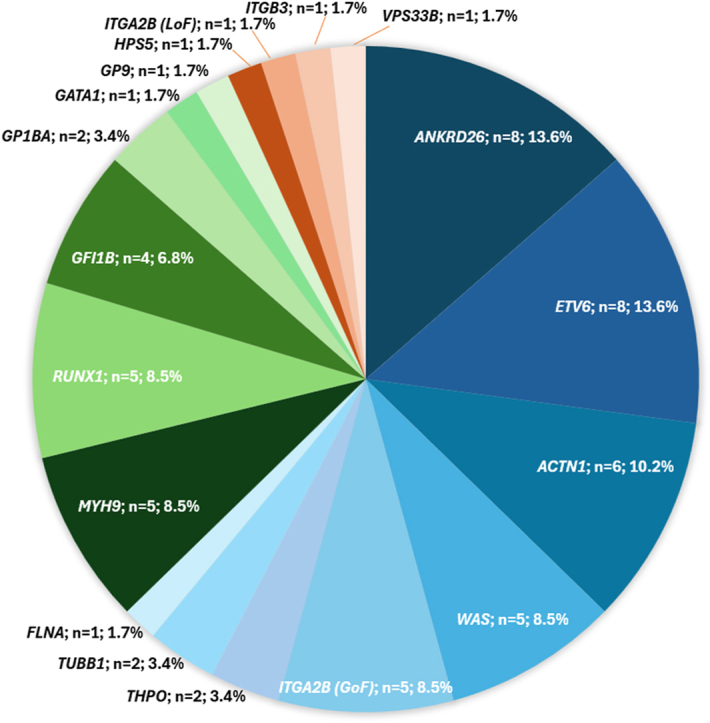

## 1 Introduction

Inherited platelet disorders (IPDs) represent a significant proportion of clinical bleeding disorders. In a registry of 26,741 patients with inherited bleeding disorders maintained by the UK Haemophilia Centres Doctors Organisation, 8% were reported to have a disorder of platelet count and/or function [1]. Despite the prevalence of such disorders among patients with clinical bleeding, the causative genetics, and thus the ultimate diagnoses, remain poorly understood. A 2014 survey by the International Society of Thrombosis and Haemostasis reported that nearly 14,000 patients were evaluated annually for suspected inherited platelet function disorders [2]. Of those evaluated, 60% had no identifiable platelet disorder, and among the 40% with identified platelet dysfunction, only 8.7% received a molecular diagnosis [2]. Whether this low rate reflected incorrect initial clinical diagnosis or limitations of the available diagnostic testing remains unclear. Regardless, the low molecular diagnostic yield in patients with known platelet defects highlights a critical need for advanced genetic diagnostic tools, as understanding the cellular and molecular pathophysiology of platelet disorders is crucial for accurate risk stratification and therapeutic personalization [3,4].

Several centers with expertise in treating patients with bleeding disorders have incorporated whole exome sequencing (WES) or targeted gene panels into their diagnostic workflow [5]. Although next-generation sequencing (NGS) is increasingly included into the workup of IPD, its application remains inconsistent and is typically utilized late in the evaluation. Functional platelet assays, such as platelet aggregation and ATP secretion, are sensitive for detecting severe platelet dysfunction but less reliable for milder qualitative platelet abnormalities and are not universally available. Moreover, platelet lumiaggregometry does not evaluate all critical aspects of platelet function for hemostasis, such as adhesion under flow, platelet-dependent thrombin generation, or thrombus contraction. Many patients lack access to functional platelet testing for reasons of platelet count, blood volume requirements, or geography. With the increasing availability and decreasing cost of NGS, it stands to reason that such testing may rise in importance in the diagnostic evaluation of IPD.

Cincinnati Children’s Hospital Medical Center (CCHMC) developed a platelet disorders gene sequencing panel targeting 67 genes known to be associated with IPDs. This testing is based on a whole exome sequence, with virtual analysis of the 67 genes of interest. From April 2018 to October 2022, 409 patients with suspected disorders of platelet function and/or platelet production were evaluated with this panel. We report our institutional experience, highlighting the panel’s diagnostic performance in establishing molecular diagnoses and identifying pathogenic variants with broader clinical implications for patients and their families. Our findings strongly support a more prominent role for NGS in the evaluation of suspected IPDs.

## 2 MethodS

### 2.1 Patient population

All patients who underwent testing with the CCHMC platelet disorders gene sequencing panel between April of 2018 and October of 2022 were reviewed. This study was approved by CCHMC institutional review board. The patient’s clinical phenotypes and other testing results, including deletion/duplication analysis by comparative genomic hybridization and family studies, were obtained through review of test requisitions and/or medical record.

### 2.2 Gene selection and panel creation

For the reported period, a literature review of genes associated with IPD had yielded a panel of 67 genes implicated in defects of platelet production or function (Supplementary Table 1), including the following: *ABCG5*, *ABCG8*, *ACBD5*, *ACTN1*, *ANKRD26*, *ANO6*, *AP3B1*, *AP3D1*, *ARPC1B*, *BLOC1S3*, *BLOC1S6*, *CD36*, *CYCS*, *DIAPH1*, *DTNBP1*, *ETV6*, *FERMT3*, *FLI1*, *FLNA*, *FYB1*, *GATA1*, *GFI1B*, *GP1BA*, *GP1BB*, *GP6*, *GP9*, *HOXA11*, *HPS1*, *HPS3*, *HPS4*, *HPS5*, *HPS6*, *ITGA2*, *ITGA2B*, *ITGB3*, *LYST*, *MASTL*, *MECOM*, *MPIG6B*, *MPL*, *MYH9*, *NBEA*, *NBEAL2*, *ORAI1*, *P2RX1*, *P2RY1*, *P2RY12, PLA2G4A*, *PRKACG*, *PTGS1*, *RAB27A*, *RASGRP2*, *RBM8A*, *RUNX1*, *SLFN14*, *STIM1*, *STX11*, *STXBP2*, *TBXA2R*, *TBXAS1*, *THPO*, *TUBB1*, *UNC13D*, *VIPAS39*, *VPS33B*, *VPS45*, and *WAS*.

### 2.3 Exome sequencing and data analysis

The platelet gene panel used the Agilent SureSelect Clinical Research Exome (CRE) V1 targeted sequence capture method (Agilent Technologies) or the Human Comprehensive Exome kit (Twist Bioscience) to enrich for the whole exome. The exomes were sequenced using Illumina HiSeq 2500 or NovaSeq 6000 sequencing system with paired-end reads (Illumina Inc). Sequence reads were mapped and compared with human genome build UCSC hg19. Data quality was assessed to confirm it had a minimum coverage of 20× for >95% of targets of interest at the whole exome level. Variants within coding exons and flanking sequences of the platelet panel were identified and evaluated by an in-house developed bioinformatic analysis pipeline. Variants in the promoter region of *ANKRD26* were analyzed; allele specific analysis for the 253-kb inversion and targeted analysis of the c.118-308 region in *UNC13D* (NM_199242.3) were performed as these variants have been shown to disrupt *UNC13D* transcription in hematopoietic cells [6]. Clinical significance of variants was assessed based on the standards and guidelines for the interpretation of sequence variants, recommended by American College of Medical Genetics and Association of Molecular Pathology [7]. Sanger sequencing was performed on all potential reportable variants. Each variant was reviewed by a faculty member in the Department of Human Genetics at CCHMC. This test did not detect large copy number changes or genomic rearrangements, other than the 1 noted earlier.

### 2.4 Review process

All variants identified through the platelet disorders gene sequencing panel were independently reviewed by 2 faculty members from the Division of Hematology at CCHMC, along with 1 faculty member from the Division of Human Genetics. Clinical interpretations and potential diagnoses were made based on the available clinical history and the best available evidence from the literature. Each variant was first evaluated from a genetic perspective to assess its pathogenic potential. Subsequently, the predicted pathogenicity was correlated with the patient’s clinical phenotype and/or biochemical findings. Patients in whom reported clinical and biochemical phenotypes aligned with the identified pathogenic/likely pathogenic variant(s), or in whom the variant(s) alone were sufficient to establish a diagnosis, were deemed diagnostic. Variants (pathogenic/likely pathogenic) considered highly likely to cause disease but that lack critical supporting information (eg, a family history and prior laboratory workup) were categorized as likely diagnostic. Variants known or predicted to be pathogenic but not explanatory of the patient’s phenotype were designated as pathogenic but nondiagnostic. This includes carrier status variants without clinical relevance to the presenting condition. Cases with only variant of unknown significance (VUS) detected were deemed with uncertain diagnosis. Recommendations for additional testing and/or clinical follow-up (eg, including targeted deletion/duplication analysis, familial testing, and further clinical evaluation) were provided based on the consensus of the Genetics and Hematology faculty reviewers.

## 3 Results

### 3.1 Cohort description

Between April 2018 and October 2022, 409 patients were tested. Demographic data are presented in Table 1; 118 patients were treated at CCHMC, while 291 were referrals from other institutions with varying degrees of clinical and biochemical background provided. Forty-six patients (all from outside institutions) had no clinical or laboratory testing history provided. All patients had concerns for underlying IPD. Of those with a reported history, 158 patients had a history of thrombocytopenia. Moreover, 130 patients had a bleeding phenotype ranging from bruising and petechiae to severe postoperative bleeding or spontaneous hemorrhage. Seventy-five patients with both thrombocytopenia and bleeding were noted. Eighty patients had a reported family history of thrombocytopenia, mucosal bleeding, or diagnosed platelet disorder. Reported prior testing included platelet aggregation studies in 106 patients (74 internal; 32 outside), electron microscopy (EM) in 27 patients (21 internal; 6 outside), and assessment of other coagulation components (ie, prothrombin time, partial thromboplastin time, and/or von Willebrand profiles) in 135 patients (106 internal; 29 outside).

**Table 1:** Demographics of patients tested for Platelet Gene Sequencing panel.

Demographic data	Value
Age range	1 month-77 years
Mean age (y)	15.54 ± 14.04
Sex	
Male	46 (188/409)
Female	54 (221/409)
Phenotype	
Thrombocytopenia	39 (158/409)
Bleeding	32 (130/409)
Mixed	18 (75/409)
Not provided	11 (46/409)
Source	
CCHMC	29 (118/409)
Outside referral	71 (291/409)

Values are % (*n*/*N*) unless specified.

CCHMC, Cincinnati Children’s Hospital Medical Center.

### 3.2 Diagnostic yield

All 409 patients successfully underwent testing with the platelet disorders gene sequencing panel (Table 2). In 78% (317/409) of patients, at least 1 reportable variant, including VUS, likely pathogenic, and pathogenic variants, was identified. Further, 40% of patients had multiple reportable variants identified (mean, 1.45 variants per patient; maximum of 5 variants).

**Table 2:** Result summary.

Variant data	No. of patients	Percentage of patients
Total patients	409	
No reportable variant identified	92	22
Any reportable variant identified	317	78
Multiple reportable variants identified	164	40
Results		
Diagnostic	59	14
Uncertain with possible significance	28	7
Uncertain (other)	230	56
Negative	92	22

**Figure 1:**
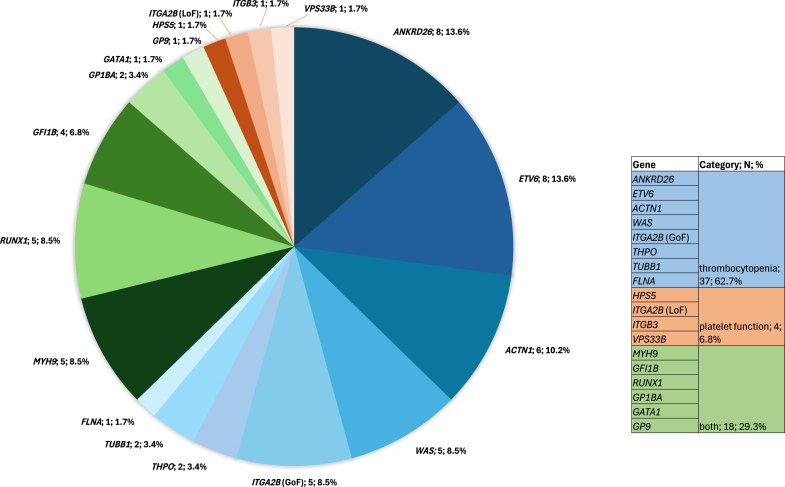
Frequency of diagnostic variants identified in the Platelet Gene Sequencing panel, grouped by gene. The table inset showed the percentage of genes and associated phenotypes within the diagnostic cohort. Genes associated with thrombocytopenia genes are shaded in blue, those related to platelet function in orange, and those affecting both in green.

Pathogenic or likely pathogenic findings consistent with the clinical phenotype were identified in 59 patients, yielding an overall diagnostic rate of 14% (Table 2). Among these, 37 (63%) cases were associated with thrombocytopenia, 4 (7%) with platelet function defects, and 18 (29%) with both thrombocytopenia and platelet dysfunction (Figure 1; Table 3, Table 4, Table 5) [[8], [9], [10], [11], [12], [13], [14], [15], [16], [17], [18], [19], [20], [21], [22], [23], [24], [25], [26], [27], [28], [29], [30], [31], [32], [33], [34], [35], [36], [37], [38], [39], [40], [41], [42], [43], [44], [45], [46], [47]]. The most frequent diagnoses were monoallelic pathogenic variants in *ANKRD26*, *ETV6*, and *ACTN1*, which are associated with thrombocytopenia, corresponding to 8 (14%), 8 (14%), and 6 (10%) cases, respectively. Other notable findings included *WAS* (5 cases, 9%), *ITGA2B* (gain of function variant) (5, 9%), *MYH9* (5, 9%), *RUNX1* (5, 9%), and *GFI1B* (4, 7%). The remaining 10 genes (*THPO*, *TUBB1*, *FLNA*, *GPP1BA*, *GATA1*, *GP9*, *HPS5*, *ITGA2B* [loss of function variants], *ITGB3*, and *VPS33B*) were represented by 1 or 2 cases each.

**Table 3:** Thirty-seven cases with molecular diagnosis of thrombocytopenia.

Patient ID	Age, sex	Clinical background	Platelets (K/μL)	Prior workup	Gene	Variant and zygosity	Associated disease	Variant classification	Previously reported?	ClinVar; gnomAD; *in silico* predictions	Diagnostic?
28	11 y, F	Unexplained thrombocytopenia	95	CBC	*ACTN1*	NM_001130004.1:c.1294G>A p.(Ala432Thr), het	Bleeding disorder, platelet-type, 15 (AD)	LP (PM1, PM2-sup, PM5, PP3)	[8]	ClinVar: 627221 (VUS) gnomAD: NA *In silico* predictions: deleterious	Yes
56	17 y, F	Unexplained thrombocytopenia; grandfather with thrombocytopenia	80	CBC	*ACTN1*	NM_001130004.1:c.2213G>A p.(Arg738Gln), het	Bleeding disorder, platelet-type, 15 (AD)	LP (PS4-mod, PM1, PM5, PM2-sup)	[9]	ClinVar: 1684471 (Path) gnomAD: NA *In silico* predictions: conflicting	Yes
38^a^	16 y, F	Unexplained thrombocytopenia; brother with thrombocytopenia and the same variant	87	CBC	*ACTN1*	NM_001130004.1:c.2255G>A p.(Arg752Gln), het	Bleeding disorder, platelet-type, 15 (AD)	LP (PS3, PS4-mod, PP1, PP3)	[10]	ClinVar: 42030 (VUS) gnomAD-all: 0.0012%; gnomAD-max (East Asian): 0.0054% *In silico* predictions: deleterious	Yes
47^a^	17 y, M	Macrothrombocytopenia, sister with macrothrombocytopenia and the same variant	NA	NA	*ACTN1*	NM_001130004.1:c.2255G>A p.(Arg752Gln), het	Bleeding disorder, platelet-type, 15 (AD)	LP (PS3, PS4-mod, PP1, PP3)	[10]	ClinVar: 42030 (VUS) gnomAD-all: 0.0012%; gnomAD-max (East Asian): 0.0054% *In silico* predictions: deleterious	Yes
37	39 y, F	Unexplained thrombocytopenia	110-130	CBC	*ACTN1*	NM_001130004.1:c.2290G>A p.(Gly764Ser), het	Bleeding disorder, platelet-type, 15 (AD)	LP (PS4-mod, PP3-mod, PP1, PP4)	[11]	ClinVar: 2581287 (VUS) gnomAD-all: 0.0004%; gnomAD-max (African American): 0.0062% *In silico* predictions: deleterious	Yes
53	14 y, M	Platelet dysfunction/defect and easy bruising; with normal whole blood platelet aggregation	Lower end of normal range	EM reportedly showed large, round platelets with decreased α granules and increased canalicular networks	*ACTN1*	NM_001130004.1:c.2290G>A p.(Gly764Ser), het	Bleeding disorder, platelet-type, 15 (AD)	LP (PS4-mod, PP3-mod, PP1, PP4)	[11]	ClinVar: 2581287 (VUS) gnomAD-all: 0.0004%; gnomAD-max (African American): 0.0062% *In silico* predictions: deleterious	Yes
3	67 y, M	Chronic thrombocytopenia with a family history affecting the father, brother, son, daughter, and grandson; also carries a diagnosis of MDS	NA	Bone marrow karyotype showed an interstitial deletion of 20q11.2q13.3 in all metaphases examined, consistent with his MDS	*ANKRD26* *RUNX1*	NM_014915.2:c.-134G>A p.(?), het NM_001754.4:c.1052_1071del p.(Gly351Aspfs∗242), somatic (VAF = 0.11)^b^	Thrombocytopenia 2 (AD) Leukemia/MDS (AD, somatic); familial platelet disorder with associated myeloid malignancy (AD)	Path (PS3, PP1-str, PS4-mod, PM1, PM2-sup, PP4) LP (PVS1-str, PM2-sup, PM5-sup)	[12] and this study	ClinVar: 30853 (Path) gnomAD: NA *In silico* predictions: NA ClinVar: NA gnomAD: NA Prediction: truncated protein that disrupts the VWRPY motif (AAs 476-480)	Yes; acquired
64	58 y, F	Thrombocytopenia and leukemia risk	NA	NA	*ANKRD26*	NM_014915.2:c.-134G>A p.(?), het	Thrombocytopenia 2 (AD)	Path (PS3, PP1-str, PS4-mod, PM1, PM2-sup, PP4)	[12]	ClinVar: 30853 (Path) gnomAD: NA *In silico* predictions: NA	Yes
55	56 y, M	Chronic thrombocytopenia with a family history in the maternal grandmother, father, and son	34-50	CBCs	*ANKRD26*	NM_014915.2:c.-126T>C p.(?), het	Thrombocytopenia 2 (AD)	LP (PS4-mod, PM1, PM5, PP1-mod, PM2-sup)	[13]	ClinVar: 626941 (Path/LP/VUS) gnomAD: NA *In silico* predictions: NA	Yes
1	2 y, F	Thrombocytopenia with family history in the mother and maternal grandfather	NA	NA	*ANKRD26*	NM_014915.2:c.-118C>A p (?), het	Thrombocytopenia 2 (AD)	Path (PS3, PS4-mod, PM1, PM5, PP1-mod, PP4, PM2-sup)	[14]	ClinVar: 812726 (LP) gnomAD: NA *In silico* predictions: NA	Yes
35	3 mo, F	Unexplained thrombocytopenia; a paternal history of a platelet disorder requiring platelet transfusion	23-61	CBCs	*ANKRD26*	NM_014915.2:c.-118C>G p.(?), het, paternally inherited	Thrombocytopenia 2 (AD)	LP (PS4-mod, PM1, PM5, PM2-sup, PP1)	[15]	ClinVar: 626940 (LP/VUS) gnomAD: NA *In silico* predictions: NA	Yes
33	1 y, M	Unexplained thrombocytopenia with a family history in the mother, maternal uncle, maternal aunt, and maternal grandfather	57	CBC	*ANKRD26*	NM_014915.2:c.-118C>T p.(?), het	Thrombocytopenia 2 (AD)	Path (PP1-str, PS4-mod, PM1, PM5, PM2-sup)	[16]	ClinVar: 626940 (Path/LP) gnomAD: NA *In silico* predictions: NA	Yes
36	47 y, F	Chronic severe thrombocytopenia with recurrent iron deficiency anemia, easy bruising, and menorrhagia requiring hysterectomy; a family history in mother and 2 maternal aunts.	NA	BM aspirate and biopsy showed megakaryocyte hyperplasia and no malignancy	*ANKRD26*	NM_014915.2:c.-118C>T p.(?), het	Thrombocytopenia 2 (AD)	Path (PP1-str, PS4-mod, PM1, PM5, PM2-sup)	[17]	ClinVar: 626940 (Path/LP) gnomAD: NA *In silico* predictions: NA	Yes
39	15 y, M	Platelet dysfunction with thrombocytopenia; a family history of bleeding in father, brother, and paternal grandmother	NA	NA	*ANKRD26*	NM_014915.2:c.-118C>T p.(?), het	Thrombocytopenia 2 (AD)	Path (PP1-str, PS4-mod, PM1, PM5, PM2-sup)	[18]	ClinVar: 626940(Path/LP) gnomAD: NA *In silico* predictions: NA	Yes
58	11 y, M	A family history of chronic unexplained thrombocytopenia	NA	NA	*ETV6*	NM_001987.4:c.1105C>T p.(Arg369Trp), likely het (VAF = 0.43)	Thrombocytopenia 5 (AD); leukemia (somatic)	LP (PM5, PP1-mod, PM2-sup, PP3)	[19]	ClinVar: 626971(Path/LP) gnomAD: NA *In silico* predictions: deleterious	Yes
46	36 y, F	Severe thrombocytopenia	NA	CBC (low WBC, elevated MCV, severe thromobocytopenia)	*ETV6*	NM_001987.4:c.1106G>A p.(Arg369Gln), likely het (VAF = 0.44)	Thrombocytopenia 5 (AD); Leukemia (somatic)	Path (PS3, PP1-str, PM5, PM2-sup, PP3)	[20]	ClinVar: 162221(Path/LP) gnomAD: NA *In silico* predictions: deleterious	Yes
44	30 y, F	Unexplained thrombocytopenia, abnormal bleeding, and easy bruising/ecchymoses	NA	NA	*ETV6*	NM_001987.4:c.1129G>C p.(Ala377Pro), likely het (VAF = 0.52)^b^	Thrombocytopenia 5 (AD); leukemia (somatic)	LP (PM1, PM5, PM2-sup, PP3)	This study Another variant Ala377Thr has been reported [19]	ClinVar: NA gnomAD: NA *In silico* predictions: deleterious	Yes
4	4 y, M	Chronic thrombocytopenia, IgA deficiency, epistaxis, easy bruising	62	Abnormal Gp1b expression by flow	*ETV6*	NM_001987.4:c.1165A>G p.(Met389Val), likely het (VAF = 0.47), de novo^b^	Thrombocytopenia 5 (AD); leukemia (somatic)	LP (PM1, PM6, PM2-sup, PP3)	This study	ClinVar: NA gnomAD: NA *In silico* predictions: deleterious	Yes
5	34 y, M	Thrombocytopenia, a family history of bleeding	NA	NA	*ETV6*	NM_001987.4:c.1196G>A p.(Arg399His), likely het (VAF = 0.53)	Thrombocytopenia 5 (AD); leukemia (somatic)	Path (PS3, PM5, PP1-mod, PP3-mod, PP2-sup)	[21]	ClinVar: 1175817 (Path/LP) gnomAD: NA *In silico* predictions: deleterious	Yes
32	13 y, M	Persistent thrombocytopenia with a history of standard-risk pre-B cell ALL; sister with chronic immune thrombocytopenia, persistent thrombocytopenia, and same variant.	NA	NA	*ETV6*	NM_001987.4:c.1196G>A p.(Arg399His), het maternally inherited	Thrombocytopenia 5 (AD); leukemia (somatic)	Path (PS3, PM5, PP1-mod, PP3-mod, PP2-sup)	[21]	ClinVar: 1175817 (Path/LP) gnomAD: NA *In silico* predictions: deleterious	Yes
62	13 y, F	Chronic thrombocytopenia and macrocytosis (without anemia) in the setting of normal vitamin B12 and folate levels	NA	Bone marrow evaluation was negative for myelodysplasia or abnormal myelopoiesis	*ETV6*	NM_001987.4:c.1196G>A p.(Arg399His), likely het (VAF = 0.50)	Thrombocytopenia 5 (AD); leukemia (somatic)	Path (PS3, PM5, PP1-mod, PP3-mod, PP2-sup)	[21]	ClinVar: 1175817 (Path/LP) gnomAD: NA *In silico* predictions: deleterious	Yes
63	29 y, F	Unexplained chronic thrombocytopenia and neutropenia	NA	NA	*ETV6*	NM_001987.4:c.1196G>A p.(Arg399His), likely het (VAF = 0.41)	Thrombocytopenia 5 (AD); leukemia (somatic)	Path (PS3, PM5, PP1-mod, PP3-mod, PP2-sup)	[21]	ClinVar: 1175817 (Path/LP) gnomAD: NA *In silico* predictions: deleterious	Yes
14	3 mo, F	Thrombocytopenia, multiple congenital brain malformations and patent ductus arteriosus	20	NA	*FLNA*	NM_001110556.1:c.6724C>T (p.Arg2242Ter), het	Thrombocytopenia with periventricular nodular heterotopia (XL)	Path (PVS1, PP1-mod, PM2-sup)	[22]	ClinVar: 405446 (Path) gnomAD: NA *In silico* predictions: loss of function	Yes
6	5 y, F	Mild thrombocytopenia with easy bruising; mother and maternal grandmother affected	79	Mean platelet volume 14 fL	*ITGA2B*	NM_000419.4:c.3076C>T p.(Arg1026Trp), het	Bleeding disorder platelet type 16 (AD); macrothrombocytopenia (AD)	Path (PS3, PP1-str, PP3, PM2-sup)	[23]	ClinVar: 50233 (Path) gnomAD: NA *In silico* predictions: deleterious	Yes
25^a^	15 y, F	Familial macrothrombocytopenia	NA	NA	*ITGA2B*	NM_000419.4:c.3076C>T p.(Arg1026Trp), het	Bleeding disorder platelet type 16 (AD); macrothrombocytopenia (AD)	Path (PS3, PP1-str, PP3, PM2-sup)	[23]	ClinVar: 50233 (Path) gnomAD: NA *In silico* predictions: deleterious	Yes
26^a^	10 y, F	Familial macrothrombocytopenia	NA	NA	*ITGA2B*	NM_000419.4:c.3076C>T p.(Arg1026Trp), het	Bleeding disorder platelet type 16 (AD); macrothrombocytopenia (AD)	Path (PS3, PP1-str, PP3, PM2-sup)	[23]	ClinVar: 50233 (Path) gnomAD: NA *In silico* predictions: deleterious	Yes
59^a^	14 y, F	Unexplained thrombocytopenia, sibling affected	NA	NA	*ITGA2B*	NM_000419.4:c.3076C>T p.(Arg1026Trp), het	Bleeding disorder platelet type 16 (AD); macrothrombocytopenia (AD)	Path (PS3, PP1-str, PP3, PM2-sup)	[23]	ClinVar: 50233 (Path) gnomAD: NA *In silico* predictions: deleterious	Yes
60^a^	13 y,F	Unexplained thrombocytopenia, sibling affected	NA	NA	*ITGA2B*	NM_000419.4:c.3076C>T p.(Arg1026Trp), het	Bleeding disorder platelet type 16 (AD); macrothrombocytopenia (AD)	Path (PS3, PP1-str, PP3, PM2-sup)	[23]	ClinVar: 50233 (Path) gnomAD: NA *In silico* predictions: deleterious	Yes
7	17 y, F	Thrombocytopenia	120	NA	*TUBB1*	NM_030773.3:c.779T>C p.(Phe260Ser), het	Macrothrombocytopenia (AD)	Path (PS3, PP3-str, PP1-mod, PM2-sup, PP4)	[24]	ClinVar: 372810 (Path/LP/VUS) gnomAD: NA *In silico* predictions: deleterious	Yes
8	13 y, F	Macrothrombocytopenia; paternal relatives affected	NA	NA	*TUBB1*	NM_030773.3:c.779T>C p.(Phe260Ser), het	Macrothrombocytopenia (AD)	Path (PS3, PP3-str, PP1-mod, PM2-sup, PP4)	[24]	ClinVar: 372810 (Path/LP/VUS) gnomAD: NA *In silico* predictions: deleterious	Yes
9	8 y, F	NA	NA	NA	*THPO*	NM_000460.3:c.13+1G>A p.(?), het^b^	Thrombocytopenia 9 (AD); amegakaryocytic thrombocytopenia, congenital, 2 (AR)	LP (PVS1, PS1-sup, PM2-sup)	This study and [25]	ClinVar: NA gnomAD: NA *In silico* predictions: disrupting splicing	Yes
2	6 y, M	Thromboctyopenia, father (platelet, 95) and paternal grandfather affected	119	Serial CBCs	*THPO*	NM_000460.3:c.469C>T p.(Arg157∗), het, paternally inherited	Thrombocytopenia 9 (AD); amegakaryocytic thrombocytopenia, congenital, 2 (AR)	Path (PVS1-str, PP1-str, PM2-sup, PP4)	[26]	ClinVar:1298946 (Path) gnomAD: NA Predictions: nonsense variant predicted to result in a truncated protein	Yes
52	6 mo, M	Abnormal bleeding and thrombocytopenia	NA	NA	*WAS*	NM_000377.2:c.134C>T p.(Thr45Met), hemi	Wiskott–Aldrich syndrome (XL); thrombocytopenia, XL	Path (PS3, PP1-str, PM6, PM2-sup, PP3)	[27]	ClinVar: 11123(Path/VUS) gnomAD: not reported *In silico* predictions: deleterious	Yes
10	10 mo, M	Thrombocytopenia, petechiae, small platelets	NA	von Willebrand testing normal	*WAS*	NM_000377.2:c.223G>A (p.Val75Met), hemi	Wiskott–Aldrich syndrome (XL); Thrombocytopenia, XL	Path (PS3, PP1-str, PM2-sup, PP3)	[28]	ClinVar: 265289 (Path) gnomAD-all: 0.0006% gnomAD-max (African): 0.0082% *In silico* predictions: deleterious	Yes
13	1 mo, M	NA	NA	NA	*WAS*	NM_000377.2:c.360+1G>A, hemi	Wiskott–Aldrich syndrome (XL); Thrombocytopenia, XL	Path (PVS1(RNA)-str, PS1, PM2-sup, PP4)	[29]	ClinVar: 381690 (Path) gnomAD: NA *In silico* predictions: disrupting splicing	Likely
15	1 y, M	Thrombocytopenia, easy bruising, eczema	NA	NA	*WAS*	NM_000377.2:c.397G>A (p.Glu133Lys), hemi	Wiskott–Aldrich syndrome (XL); thrombocytopenia, XL	Path (PS3, PM1, PM5, PM2-sup, PPS)	[27]	ClinVar: 870492 (LP) gnomAD: NA *In silico* predictions: deleterious	Yes
57	4 mo, M	Unexplained thrombocytopenia	7	CBC	*WAS*	NM_000377.2:c.1271del p.(Gly424AlafsTer21), hemi	Wiskott–Aldrich syndrome (XL); thrombocytopenia, XL	Path (PVS1, PM2-sup, PP4)	[27]	ClinVar: 2138571 (Path) gnomAD: NA Prediction: loss of function	Yes

AD, autosomal dominant; AML, acute myeloid leukemia; APTT, activated partial thromboplastin time; AR, autosomal recessive; BM, bone marrow; CBC, complete blood count; EM, electron microscopy; gnomAD, genome aggregation database v.2.1.0; hemi, hemizygous; het, heterozygous; HI, haploinsufficiency; homo, homozygous; LP, likely pathogenic; MCV, mean corpuscular volume; MDS, myelodysplastic syndrome; mod, moderate; MPV, mean platelet volume; NA, not available; Path, pathogenic; PT, prothrombin time; PTT, partial thromboplastin time; RCoF, ristocetin cofactor; str, strong; sup, supporting; VAF, variant allele fraction; VUS, variant of unknown significance; VWF, von Willebrand factor; XL, X-linked; XLR, X-linked recessive.

a Patients 25 and 26 are full siblings. Patients 38 and 47 are fraternal twins. Patients 59 and 60 are full siblings.

b Previously unreported variants.

**Table 4:** Four cases with molecular diagnosis of platelet function defect.

Patient ID	Age, sex	Clinical background	Platelet (K/μL)	Prior workup	Gene	Variant	Associated disease	Variant classification	Previously reported?	ClinVar; gnomAD; *in silico* predictions	Diagnostic?
61	51 y, F	Symptoms suspicious for Hermansky–Pudlak syndrome	NA	NA	*HPS5*	NM_181507.1:c.1165-1G>A p.(?), het^a^ NM_181507.1:c.1423del p.(Leu475SerfsTer37), het	Hermansky–Pudlak syndrome 5 (AR)	LP (PVS1-strong, PM2-sup, PM3-sup) Path (PVS1, PM2-sup, PM3-sup)	This study and [30]	ClinVar: 2022312 (LP) gnomAD-all: 0.0008% gnomAD-max (European—non-Finnish): 0.0018% *In silico* predictions: disrupting splice site ClinVar: 431164 (Path/LP/VUS) gnomAD-all: 0.0080% gnomAD-max (Ashkenazi Jewish): 0.1687% Prediction: loss of function	Likely (unknown phase)
84	21 y, M	Abnormal bleeding, excessive bruising, hematochezia, microcytic anemia and a history of Glanzmann thrombasthenia	NA	NA	*ITGA2B*	NM_000419.4:c.1553T>A p.(Ile518Asn), het NM_000419.4:c.1946+3G>T p.(?), het	Glanzmann thrombasthenia 1 (AR)	LP (PP4-str, PM2-sup, PM3-sup, PP3) LP (PP4-str, PM2-sup, PM3-sup)	[31]	ClinVar: 2498358(LP) gnomAD-all: 0.0004% gnomAD-max (South Asian): 0.0009% *In silico* predictions: deleterious ClinVar: 2498360(LP) gnomAD: NA *In silico* predictions: disrupt the nearby splice donor site	Likely (unknown phase)
34	11 mo, F	A history of easy bruising	Elevated	von Willebrand testing did not show evidence of disease	*ITGB3*	NM_000212.2:c.100C>T p.(Arg34Ter), het, paternally inherited NM_000212.2:c.629G>A p.(Cys210Tyr), het, maternally inherited	Glanzmann thrombasthenia (AR)	Path (PVS1, PM3, PM2-sup) Path (PS3, PM3, PP4-mod, PM2-mod, PP1, PP3)	[32,33]	ClinVar: 996193 (Path) gnomAD-all: 0.0018% gnomAD-max (African American): 0.0080% *In silico* predictions: loss of function ClinVar: NA gnomAD: NA *In silico* predictions: deleterious	Yes
29	14 y, F	A history of platelet dysfunction	Normal	CBC; whole blood platelet aggregometry showed absent response to ADP and thromboxane A2 analog and impaired ATP secretion in response to ADP and collagen	*VPS33B*	NM_018668.4:c.1225+5G>C p.(?) het Heterozygous partial gene deletion of *VPS33B* (exon1-intron1)^a^^,^^b^ arr[hg19] 15q26.1(91564827_91567692)x1^a^	Arthrogryposis, renal dysfunction, and cholestasis 1 (AR)	Path (PVS1(RNA)-str,PS3, PM3, PM2-sup, PP4) LP (PVS1, PM2-sup)	[34] and this study	ClinVar: 88858(Path/LP) gnomAD-all: 0.0020%; gnomAD-max (Admix American): 0.0116% *In silico* prediction: disrupt the nearby splice donor ClinVar: NA gnomAD: NA Prediction: loss of function	Likely (unknown phase)

AD, autosomal dominant; AML, acute myeloid leukemia; APTT, activated partial thromboplastin time; AR, autosomal recessive; BM, bone marrow; CBC, complete blood count; EM, electron microscopy; gnomAD, genome aggregation database v.2.1.0; hemi, hemizygous; het, heterozygous; HI, haploinsufficiency; homo, homozygous; LP, likely pathogenic; MCV, mean corpuscular volume; MDS, myelodysplastic syndrome; mod, moderate; MPV, mean platelet volume; NA, not available; Path, pathogenic; PT, prothrombin time; PTT, partial thromboplastin time; RCoF, ristocetin cofactor; str, strong; sup, supporting; VAF, variant allele fraction; VUS, variant of unknown significance; VWF, von Willebrand factor; XL, X-linked; XLR, X-linked recessive.

a Previously unreported variants.

b Deletion between 2.87 and 20.61 Kb. Minimum breakpoints: g.91564827-g.91567692. Maximum breakpoints: g.91563805-g.91584416. This deletion was detected in the follow-up deletion/duplication comparative genomic hybridization test.

**Table 5:** Eighteen cases with molecular diagnosis of platelet function defect and thrombocytopenia.

Patient ID	Age, sex	Clinical background	Platelet (K/μL)	Prior workup	Gene	Variant	Associated disease	Variant classification	Previously reported?	ClinVar; gnomAD; *in silico* predictions	Diagnostic?
48	2 y, M	Unexplained thrombocytopenia	NA	NA	*GATA1*	NM_002049.3:c.647G>A p.(Arg216Gln), hemi	Thrombocytopenia, XL, with or without dyserythropoietic anemia (XLR); thrombocytopenia with β-thalassemia, XL (XLR); leukemia, megakaryoblastic, with or without Down syndrome, somatic	Path (PS3, PP1-str, PM5, PM2-sup, PP3)	[35]	ClinVar: 10428 (Path) gnomAD: NA *In silico* predictions: deleterious	Yes
21	13 y, F	Thrombocytopenia; mother (platelet, 126) and brother (platelet, 105) affected, both carrying the same variant	63	NA	*GFI1B*	NM_004188.6:c.503G>T p.(Cys168Phe), het, maternally inherited	Bleeding disorder, platelet-type, 17 (AD/AR)	LP (PS3, PP1-mod, PP3)	[36]	ClinVar: NA gnomAD-all: 0.0490% gnomAD-max (South Asian): 0.3955% *In silico* predictions: deleterious	Yes
50	17 y, F	Abnormal bleeding, unexplained thrombocytopenia, and a family history of bleeding	131	CBC	*GFI1B*	NM_004188.6:c.503G>T p.(Cys168Phe), het	Bleeding disorder, platelet-type, 17 (AD/AR)	LP (PS3, PP1-mod, PP3)	[36]	ClinVar: NA gnomAD-all: 0.0490% gnomAD-max (South Asian): 0.3955% *In silico* predictions: deleterious	Yes
41	9 y, M	Unexplained thrombocytopenia	NA	NA	*GFI1B*	NM_004188.6:c.503G>T p.(Cys168Phe), homo	Bleeding disorder, platelet-type, 17 (AD/AR)	LP (PS3, PP1-mod, PP3)	[36]	ClinVar: NA gnomAD-all: 0.0490% gnomAD-max (South Asian): 0.3955% *In silico* predictions: deleterious	Yes
27	20 mo, F	Thrombocytopenia; father and sister affected, both carrying the same variant	108	CBC	*GFI1B*	NM_004188.6:c.859C>T p.(Gln287Ter), het, paternally inherited	Bleeding disorder, platelet-type, 17 (AD/AR)	Path (PVS1, PP1-str, PM2-sup, PP4)	[37]	ClinVar: 102428 (Path) gnomAD: NA Prediction: loss of function	Yes
51	19 y, F	Easy bleeding and bruising, thrombocytopenia and platelet dysfunction	74	Increased immature platelet fraction and absolute platelet fraction	*GP1BA*	NM_000173.6:c.217C>T p.(Leu73Phe), het	Bernard–Soulier, type A2 (AD)	LP (PS4-mod, PP1-mod, PM2-sup, PP4)	[38]	ClinVar: 4154 (LP) gnomAD: NA *In silico* predictions: conflicting	Yes
22	3 mo, M	Thrombocytopenia, father, paternal uncle, paternal aunt, paternal grandfather, and paternal great grandfather affected	133	CBC	*GP1BA*	NM_000173.6:c.521A>G p.(Asn174Ser), het, paternally inherited^a^	Bernard–Soulier, type A2 (AD)	LP (PM1, PM2-sup, PP1, PP3, PP4)	Not reported; however, nearby variant, Ala172Val is pathogenic [39]	ClinVar: NA gnomAD: NA *In silico* predictions: deleterious	Yes
54	1 y, F	Congenital macrothrombocytopenia; aunt with thrombocytopenia	NA	NA	*GP9*	NM_000174.4:c.182A>G p.(Asn61Ser), homo	Bernard–Soulier syndrome, type C (AR)	Path (PP1-str, PM3, PP4-mod, PS3-sup, PP3)	[40]	ClinVar: 13529 (Path/LP) gnomAD: NA *In silico* predictions: deleterious	Yes
24	5 y, F	Thrombocytopenia, easy bruising, cataracts	1-3	CBC	*MYH9*	NM_002473.5:c.287C>T p.(Ser96Leu), het	Macrothrombocytopenia and granulocyte inclusions with or without nephritis or sensorineural hearing loss (AD)	Path (PS3, PM6-str, PM2-sup, PP3)	[41]	ClinVar: 14083 (Path) gnomAD: NA *In silico* predictions: deleterious	Yes
30	17 y, F	Incidentally discovered thrombocytopenia with heavy menstrual bleeding; a family history of thrombocytopenia and excessive bleeding	88	Döhle bodies in the neutrophils	*MYH9*	NM_002473.5:c.3493C>T p.(Arg1165Cys), het	Macrothrombocytopenia and granulocyte inclusions with or without nephritis or sensorineural hearing loss (AD)	Path (PP1-str, PM6, PM5, PM2-sup, PP3)	[42]	ClinVar: 14074 (Path) gnomAD: NA *In silico* predictions: deleterious	Yes
31	17 y, F	Thrombocytopenia; diagnosed with May–Hegglin anomaly/macrothrombocytopenia with bleeding symptoms; father affected	20-30	Neutrophil inclusions	*MYH9*	NM_002473.5:c.5521G>A p.(Glu1841Lys), het	Macrothrombocytopenia and granulocyte inclusions with or without nephritis or sensorineural hearing loss (AD)	Path (PS3, PS4, PM2-sup, PP3)	[43]	ClinVar: 14073 (Path/LP) gnomAD: NA *In silico* predictions: deleterious	Yes
40	9 mo, M	Not provided	NA	NA	*MYH9*	NM_002473.5:c.5521G>A p.(Glu1841Lys), het	Macrothrombocytopenia and granulocyte inclusions with or without nephritis or sensorineural hearing loss (AD)	Path (PS3, PS4, PM2-sup, PP3)	[43]	ClinVar: 14073 (Path/LP) gnomAD: NA *In silico* predictions: deleterious	Yes
23	23 mo, F	Macrothrombocytopenia, Döhle bodies on smear, no bleeding; mother with *MYH9* variant	84	CBC	*MYH9* *GP9* *ABCG8*	NM_002473.5:c.5521G>A (p.Glu1841Lys), het NM_000174.3:c.182A>G (p.Asn61Ser), het NM_022437.2:c.1083G>A (p.Trp361∗), het	Macrothrombocytopenia and granulocyte inclusions with or without nephritis or sensorineural hearing loss (AD) Bernard–Soulier, type C (AR) Sitosterolemia (AR)	Path (PS3, PS4, PM2-sup, PP3) Path (PP1-str, PM3, PP4-mod, PS3-sup, PP3) Path (PVS1, PM3, PM2-sup)	[[43], [44], [45]]	ClinVar: 14073 (Path/LP) gnomAD: NA *In silico* predictions: deleterious ClinVar: 13529 (Path/LP) gnomAD-all: 0.0508% gnomAD-max (European–non-Finnish): 0.0949% *In silico* predictions: deleterious ClinVar: 4967 (Path) gnomAD-all: 0.0937% gnomAD-max (European–Finnish): 0.2547% *In silico* predictions: null	Yes uncertain (second variant not detected) Uncertain (second variant not detected)
42	21 y, F	A history of easy bruising, heavy menstrual bleeding, and moderate thrombocytopenia, with a suspected functional platelet defect attributed to dense granule disorder	NA	Platelet-rich plasma aggregation and functional testing showed reduced maximal aggregation and ATP secretion in response to all agonists except ristocetin. Quinacrine uptake and release were abnormal.	*RUNX1*	NM_001754.4:c.553C>T p.(Gln185Ter), het, germline variant confirmed in tissue and blood^a^	Platelet disorder, familial, with associated myeloid malignancy (AD); leukemia, acute myeloid (AD, somatic)	Path (PVS1, PM2-sup, PM5_sup)	This study	ClinVar: NA gnomAD: NA Prediction: in exon 6 of 9, predicted to result in loss of function through NMD	Yes
49	19 y, M	Not provided	NA	NA	*RUNX1*	NM_001754.4:c.814C>T p.(Gln272Ter), likely het (VAF = 0.54)^a^	Platelet disorder, familial, with associated myeloid malignancy (AD); leukemia, acute myeloid (AD, somatic)	Path (PVS1, PM2-sup, PM5-sup)	This study	ClinVar: 2123057 (Path) gnomAD: NA Prediction: in exon 8 of 9, predicted to result in loss of function through NMD or truncated protein, which disrupts the VWRPY motif (Aas476-480)	Likely
43	5 y, F	Unexplained thrombocytopenia	NA	NA	*RUNX1*	NM_001754.4:c.1210del p.(His404ThrfsTer190), likely het (VAF = 0.51)^a^	Platelet disorder, familial, with associated myeloid malignancy (AD); leukemia, acute myeloid (AD, somatic)	LP (PVS1-str, PM2-sup, PM5-sup)	This study	ClinVar: 2730693 (LP) gnomAD: NA Prediction: truncated protein, which disrupts the VWRPY motif (AAs 476-480)	Yes
45	9 y, M	Unexplained thrombocytopenia	NA	NA	*RUNX1*	NM_001754.4:c.1242C>G p.(Tyr414Ter), likely het (VAF = 0.56)	Platelet disorder, familial, with associated myeloid malignancy (AD); leukemia, acute myeloid (AD, somatic)	Path (PVS1-str, PS4, PM2-sup, PM5-sup, PP1)	[46]	ClinVar: 1194557 (LP) gnomAD: NA Prediction: truncated protein, which disrupts the VWRPY motif (AAs 476-480)	Yes
87	14 mo, M	Thrombocytopenia since birth, with a maternal history of chronic thrombocytopenia	10-86	CBC	*RUNX1*	Heterozygous whole-gene deletion of *RUNX1,* maternally inherited^b^ arr[hg19] 21q22.11q22.12(35305042_36861310)x1 mat	Platelet disorder, familial, with associated myeloid malignancy (AD); leukemia, acute myeloid (AD, somatic)	Path (ClinGen HI Score: 3 (PVS1, PM6, PM2-sup, PP1)	[47]	ClinGen HI Score: 3 (sufficient evidence for haploinsufficiency) gnomAD: NA	Yes

AD, autosomal dominant; AML, acute myeloid leukemia; APTT, activated partial thromboplastin time; AR, autosomal recessive; BM, bone marrow; CBC, complete blood count; EM, electron microscopy; gnomAD, genome aggregation database v.2.1.0; hemi, hemizygous; het, heterozygous; HI, haploinsufficiency; homo, homozygous; LP, likely pathogenic; MCV, mean corpuscular volume; MDS, myelodysplastic syndrome; mod, moderate; MPV, mean platelet volume; NA, not available; Path, pathogenic; PT, prothrombin time; PTT, partial thromboplastin time; RCoF, ristocetin cofactor; str, strong; sup, supporting; VAF, variant allele fraction; VUS, variant of unknown significance; VWF, von Willebrand factor; XL, X-linked; XLR, X-linked recessive.

a Previously unreported variants.

b Deletion between 1.56 and 1.59 Mb. Minimum breakpoints: g.35305042-g.36861310. Maximum breakpoints: g.35277893-g.36872344. This deletion was detected in the follow-up deletion/duplication comparative genomic hybridization test.

### 3.3 Uncertain diagnosis may be clarified through follow-up analysis

**Figure 2:**
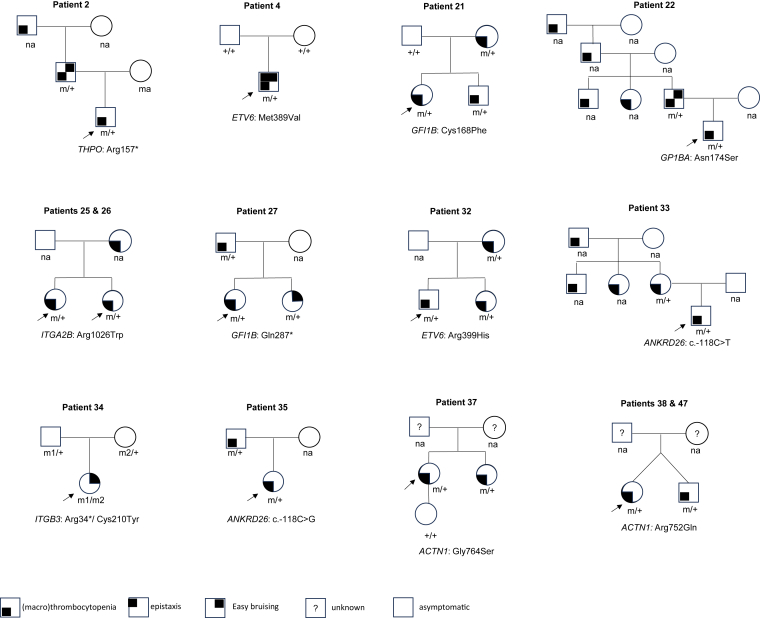
Family study results. Black arrows indicate probands. Circles represent females, squares represent males. The legend for symbols and keys is provided at the bottom of the figure. Patients 38 and 47 are fraternal twins.

A significant number of cases (258 cases, 63%) had uncertain diagnoses. Among these, 28 cases were considered highly likely clarifiable with additional follow-up to assess their relevance to the patient’s phenotype (Supplementary Table 2). These cases fell into the following categories: (1) patients with a single heterozygous pathogenic or likely pathogenic variant in a gene associated with a recessive condition, where a second variant was not detected; (2) individuals with biallelic VUS in a gene associated with a recessive condition; (3) patients hemizygous for a VUS in a gene associated with an X-linked condition; and (4) patients heterozygous for a VUS in a gene associated with a dominant condition. To further clarify the pathogenicity and clinical significance of these variants, additional investigations are warranted, such as clinical correlation, segregation analysis through family studies, deletion/duplication testing to assess the second allele, RNA sequencing to evaluate splicing/expression, noncoding region sequencing to identify deep intronic or untranslated region (UTR) variants, or other clinically available platelet function tests. Figure 2 shows 12 representative family study results from this cohort (21 in total), which provided additional evidence for variant pathogenicity.

Highlighting the utility of recommended follow-up testing, a 3-month-old boy (Table 5 and Figure 2, patient 22) presented with mild pancytopenia including thrombocytopenia (platelet count of 133,000/μL) and a notable paternal family history of thrombocytopenia (with father, uncle, aunt, grandfather, and great grandfather affected). Genetic testing revealed a heterozygous missense variant in *GP1BA*, c.521A>G p.(Asn174Ser), resulting in a substitution of a well conserved asparagine with serine at amino acid position 174 in the leucine-rich repeats region of the von Willebrand factor–binding domain of glycoprotein Ibα. Although this specific variant has not been previously reported, a nearby pathogenic missense variant, c.515C>T p.(Ala172Val), is a well-documented cause of autosomal dominant Bernard–Soulier syndrome and is known as the most frequent cause of inherited thrombocytopenia in Italy [39,[48], [49], [50]], suggesting the functional importance of this region. Follow-up familial-targeted genetic testing confirmed that this variant was paternally inherited, supporting its potential pathogenicity and consistent with the observed inheritance pattern.

Another example is a 4-year-old boy (Table 3 and Figure 2, patient 4) with a history of mild thrombocytopenia, petechiae, and mucosal bleeding. Prior (outside) testing revealed IgA deficiency and a heterozygous missense VUS in *TUBB1*, which is also carried by his asymptomatic father who had normal platelet counts. The platelet disorders gene sequencing panel identified the aforementioned *TUBB1* variant, as well as a novel variant in *ETV6*, c.1165A>G p.(Met389Val), which was initially interpreted as a VUS based on available evidence, although possibly explaining his phenotype. Subsequent targeted analyses for this variant in both parents showed neither of them was a carrier, suggesting it is a de novo variant (Figure 2). Therefore, the *ETV6* p.(Met389Val) variant was upgraded to likely pathogenic. He subsequently underwent bone marrow aspirate and biopsy, which showed normal trilineage hematopoiesis without evidence of malignancy. Monitoring with serial complete blood counts was recommended.

### 3.4 Hematologic malignancy risk

In addition to providing a potential explanation for patients’ platelet phenotypes, several identified variants were in genes also associated with familial myelodysplastic syndrome (MDS) and hematologic malignancy, similar to the case described earlier (patient 4). Twenty-two cases (37% of all diagnostic cases) carried such variants, including 8 in *ETV6*, 8 in *ANKRD26*, and 6 in *RUNX1*, 1 of which with a gross full gene deletion (Tables 3 and 5).

Variants in *ETV6* and *RUNX1* may be acquired (somatic) or constitutional (germline/inherited) in origin. Because the platelet disorders gene sequencing panel cannot distinguish between somatic or germline variants with certainty, testing of nonhematopoietic tissue (eg, cultured fibroblasts) is recommended to clarify variant origin. For example, in patient 42 in Table 5, the *RUNX1* nonsense variant, p.(Gln185Ter), was confirmed in both blood-derived and skin-derived DNA, consistent with a germline variant.

All pathogenic or likely pathogenic *RUNX1* variants detected were either gross deletions or nonsense/frameshift variants predicted to result in loss of function through nonsense-mediated mRNA decay or production of a truncated protein lacking the C-terminal VWRPY motif (amino acids 476-480) (Tables 3 and 5). Four of these *RUNX1* variants have not been previously reported in literature. The VWRPY motif is critical for RUNX1-mediated regulation of hematopoietic progenitor maturation and megakaryocyte differentiation [[51], [52], [53]]. Four of 6 *RUNX1* variants identified in this cohort were located in the C-terminal region and predicted to abolish this functional motif.

A 67-year-old man with chronic thrombocytopenia and a strong family history of thrombocytopenia (in father, brother, son, daughter, and grandson) was also diagnosed with MDS (Table 3, patient 3). Two pathogenic variants, one in *ANKRD26* and another in *RUNX1*, were identified. He was heterozygous for a previously reported 5'UTR pathogenic variant, c.-134G>A, in *ANKRD26,* and also carried a previously unreported, likely somatic *RUNX1* frameshift variant, with variant allele fraction (VAF) of 11%, c.1052_1071del p.(Gly351Aspfs∗242). This *RUNX1* variant removes the final 130 amino acids, replaces them with 241 aberrant residues, and disrupts the VWRPY motif, consistent with a pathogenic classification. Reportedly, his bone marrow chromosome karyotype analysis (outside) revealed an interstitial deletion of 20q11.2q13.3 in all metaphases examined, a recurrent finding in MDS. The *ANKRD26* variant likely accounts for the familial thrombocytopenia, while the *RUNX1* variant is consistent with clonal evolution toward MDS. Indeed, *RUNX1* variants are recognized as main molecular predictors of rapid progression in MDS and are important for both prognostic and therapeutic stratification [54].

### 3.5 Multiple system disorders

The platelet disorders gene sequencing panel contains genes associated with qualitative and quantitative platelet disorders as part of the phenotypic spectrum. Mild bleeding symptoms may represent the initial manifestation of a more serious, possibly multisystemic, underlying condition [4]. For example, 5 cases were identified with pathogenic variants in *MYH9* (Table 5), a gene associated with a spectrum of autosomal dominant macrothrombocytopenia characterized by variable degrees of thrombocytopenia, platelet macrocytosis, Döhle-like inclusions in leukocytes, and, in some cases, progressive nephritis, sensorineural hearing loss, or cataracts. The identification of a specific pathogenic *MYH9* variant is therefore clinically meaningful, as it enables relevant anticipatory guidance, targeted surveillance, and early intervention to prevent or mitigate multisystem complications. Precise genetic characterization thus not only clarifies disease etiology of platelet defects but also informs longitudinal care.

## 4 Discussion

Achieving a molecular diagnosis in patients with suspected IPD has historically been challenging, and diagnostic rates remain low, with reported rates of only 8.7% [2]. Lack of a specific diagnosis forces clinicians to manage bleeding phenotypes without optimal information for treatment or prognostic planning and denies patients and families crucial knowledge for genetic counseling and family planning.

NGS has transformed the diagnostic landscape across a wide spectrum of genetic disorders as sequencing costs decrease and efficiency increases. Despite this progress, NGS remains a late adjunct in the diagnostic algorithms for IPDs [1,2]. The ideal role and timing of NGS in IPD evaluation are still being explored by many centers, including CCHMC. As platelet disorders represent a distinct subset of bleeding disorders for which functional laboratory testing is imperfect and often not feasible, we developed a robust, platelet-specific gene panel to enable high-yield, targeted testing that is beyond the scope of commercially available broad hemostasis gene panels. In this study, we present our 5-year experience with a large cohort of pediatric and adult patients tested using our platelet disorders gene sequencing panel. The overall rate of molecular diagnosis was 14%. Although direct comparisons are limited by differences in cohort size and gene content among studies, our results are consistent with previously reported diagnostic rate using targeted gene panels for IPD evaluation [5,[55], [56], [57]].

A notable subset of patients (22, 37%) are those harboring variants with the World Health Organizatrion classification of myeloid neoplasms with germline predisposition [58], including 8 in *ETV6*, 8 in *ANKRD26*, and 6 in *RUNX1*. Germline *ETV6* variants and their dual association with IPD and hematologic malignancy were not fully appreciated until 2015 [20]. Other than mild thrombocytopenia, decreased platelet aggregation to ADP and arachidonic acid, normal platelet ultrastructure, ∼20% to 30% of individuals with *ETV6* germline pathogenic variants develop a hematologic malignancy [59,60]. Functionally, *ETV6* variants disrupt DNA binding and nuclear localization, impairing bone marrow colonization by progenitor cells and primitive hematopoiesis (particularly in megakaryocytes), thereby predisposing to both thrombocytopenia and hematopoietic malignancy [60]. Germline *ANKRD26* variants confer a ∼8% to 10% lifetime risk of hematologic malignancy, typically with a later onset (35-70 years) [61,62]. Although the mechanisms remain incompletely defined, pathogenic variants in the 5' UTR of *ANKRD26* disrupt binding to *RUNX1* and *FLI1*, thereby impairing megakaryopoiesis and bone marrow function [60]. *ANKRD26-*related thrombocytopenia generally presented with mild thrombocytopenia, reduced α granules, and cytoplasmic inclusions on EM [60]. *RUNX1* encodes a transcription factor crucial for hematopoiesis, and pathogenic variants impair megakaryocyte maturation, T and B lymphocyte development, and regulation of hematopoietic progenitors [60]. The association between platelet dysfunction and myeloid malignancy was first described in 1978 in families with impaired aggregation responses to epinephrine and arachidonic acid [63] and later attributed to germline *RUNX1* variants [60]. Although these variants alone are insufficient for leukemogenesis, secondary somatic events frequently result in malignant transformation, with a 30% to 40% lifetime risk of MDS and/or acute myeloid leukemia and a median onset age of 33 years [60,64,65]. Specific to platelet lineage, *RUNX1* variants typically cause mild to moderate thrombocytopenia with reduced dense granules, decreased aggregation to epinephrine, and diminished fibrinogen binding [60].

Variants in *ETV6* and *RUNX1* may be somatic or inherited in origin. Somatic *ETV6* variants have been identified in 1.5% of hematologic malignancies overall, occurring in 4% of chronic lymphocytic leukemia, 3% of MDS, and 2% of B cell acute lymphoblastic leukemia, and are associated with poor prognostic in MDS [66,67]. Similarly, somatic *RUNX1* variants have been reported in 10% of acute myeloid leukemia, 12% of MDS, 2% to 4% of myeloproliferative neoplasms, 15% of chronic myelomonocytic leukemia, and 2% to 4% of clonal cytopenias of unclear significance [64]. Testing of nonhematopoietic tissue (eg, cultured fibroblasts) can help clarifying variant origin.

The question of whether to test asymptomatic individuals for variants with predisposition to myeloid malignancies remains controversial. Advocates highlight the benefits of early detection, the opportunity for surveillance and intervention, and the value of identifying at-risk relatives for genetic counseling. Detractors note that early diagnosis rarely alters outcomes and may increase patient anxiety, given the lack of preventive therapy. Weighing both sides, our group supports testing such variants in patients with IPD after obtaining informed consent, given the substantial malignancy risks and the clinical and familial value of molecular information. Our approach in individuals identified with such variants has been to offer information about potential risk and benefits of screening strategies, such as complete blood counts or yearly bone marrow aspirates and biopsies, particularly as the patients age. Malignancy in these disorders generally presents in or beyond the second and third decades of life, and, for many patients at risk for myeloid malignancies, the chance of cure is higher if the myelodysplasia is detected before frank leukemia develops. Furthermore, these results underscore the critical role of genetic counseling for family planning and long-term health maintenance.

Other variants identified in our cohort, although not associated with malignancy, highlight the utility of a precise molecular diagnosis in diseases with diverse phenotypic manifestations. Patient 23 (Table 5), a 23-month-old girl with macrothrombocytopenia, Döhle bodies, and a maternal history of May–Hegglin anomaly, was found to have a heterozygous *MYH9* c.5521G>A p.(Glu1841Lys) variant. Variants in *MYH9* cause a spectrum of diseases now collectively referred to as *MYH9*-related disease but were previously clinically categorized as May–Hegglin anomaly, Sebastian syndrome, Epstein syndrome, Fechtner syndrome, and autosomal dominant deafness. The variable risks and severity of thrombocytopenia, nephritis, sensorineural hearing loss, and cataracts, which often evolve over time [68], depend at least in part on the location of the variant within certain regions. Pathogenic variants in the globular head domain often result in more severe thrombocytopenia. Location within the tail domain can be associated with milder thrombocytopenia and lower risk of nephritis and deafness. This patient’s heterozygous *MYH9* c.5521G>A p.(Glu1841Lys) variant is located at myosin tail domain and has been associated with hearing loss, nephritis, and cataracts [68]. Identification allowed early initiation of screening for complications and timely referral to subspecialists for the patient and for the mother.

Patient 61 (Table 3) had identified variants associated with Hemansky–Pudlak syndrome, another multisystem disorder not only typically presenting with oculocutaneous albinism and absent dense granules on platelet EM but also linking to life-threatening pulmonary fibrosis [69]. These examples, although not exhaustive, illustrate how molecular precision directly informs clinical management and highlights the need for early genetic evaluation in patient with IPD.

The presence and frequency of variants with implications beyond platelet function raise an important and sometimes controversial question regarding appropriate follow-up and counseling. At CCHMC, it is standard practice for the treating hematologist to initiate the informed consent process prior to ordering genetic testing and then to discuss results in detail with the patient and family. These discussions include interpretation of the findings, their known and potential multisystem impact, and recommendations for further testing (such as deletion/duplication testing or familial testing). Risks and benefits of disease monitoring, if relevant, are reviewed and suggestions for screening can be tailored to the specific variant, as for patient 23 with the *MYH9* variant. Shared decision making with the family and the patient is important, with individualized follow-up, based on patient/family preference and genetic variant discovered. Identification of deleterious variants also enables family planning and genetic counseling.

While the overall diagnostic rate of our IPD gene panel represents a significant improvement over conventional diagnostic tools, there remains considerable room for improvement. Multiple factors likely contribute to the continued diagnostic conundrum in IPD. One major challenge is the high prevalence of VUS throughout the cohort, reflecting both our incomplete understanding of the genome and the complex genetic regulation of platelet biology. Reporting novel variants, whether pathogenic or VUS, to publicly accessible databases such as ClinVar is essential for advancing the collective understanding of IPD genetics. It is also notable that the majority of the pathogenic or likely pathogenic variants identified involved abnormalities in platelet number. Many patients in our cohort exhibited clear platelet dysfunction without any causative variants or acquired causes of dysfunction identified, suggesting that additional IPD-related genes involved in platelet function remain undiscovered.

One major strength of the exome-based virtual gene panel is its scalability. Because the full exome is sequenced but only selected genes are analyzed, the test can be seamlessly expanded as new IPD-associated genes are identified. Additionally, the underlying exome data can be reanalyzed as knowledge evolves. It could also be mined retrospectively for research after appropriate institutional review board approval.

Nonetheless, this approach has inherent limitations. As a targeted virtual gene panel, it is still subject to periodic updates as new disease-associated genes are identified. In addition, WES may miss clinically relevant variants in regions with low coverage, noncoding regions, or areas with homologous sequences, and our assay does not assess large copy number variants or genomic rearrangements. Therefore, the absence of identified pathogenic variants does not exclude genetic etiology for the patient’s symptoms.

Complementary tests, such as deletion/duplication analysis by comparative genomic hybridization, are crucial adjuncts for comprehensive evaluation. Additional testing strategies, including parental targeted analyses to see whether the variant is de novo or inherited, variant phasing to establish *cis*- or *trans* configuration, and segregation studies to assess variant cosegregation with disease in families, provide valuable evidence to refine variant classification (Figure 2). Unfortunately, these analyses were underused in our cohort, where only 21 families pursued such testing. Expanded implementation of these follow-up studies could substantially improve diagnostic accuracy and variant classification in future evaluations.

After this cohort was analyzed, our laboratory changed the sequencing platform for the platelet disorders gene sequencing panel from whole exome to whole genome sequencing. This strategy will better interrogate noncoding regions of the genome and is more sensitive to large genomic rearrangements and copy number changes. However, interpretation of whole genome sequencing data remains challenging, as normal variation in noncoding regions is not yet well defined. Over time, as the knowledge of variants in these regions increases, this expanded approach is expected to yield higher diagnostic rates for IPDs.

## 5 Conclusion

Despite the existence of algorithmic guidelines, access to sophisticated functional testing and diagnostic approaches for patients with suspected IPD remain highly variable. Genetic testing, such as by NGS, is often reserved for late stages of evaluation, and insurance reimbursement for genetic testing is often challenging to secure. Review of our institutional experience using an exome-based virtual gene panel approach demonstrated important improved diagnostic yield, although not universal diagnostic resolution. Importantly, the implications of sequencing results frequently extended well beyond IPD, providing patients and families with valuable information for clinical management, surveillance, family planning, and genetic counseling. Earlier incorporation of NGS into the diagnostic pathway could facilitate timely identification of variants with drastic implications, potentially altering clinical outcomes. As sequencing becomes more accessible and affordable, its role is likely to shift toward to the forefront of IPD evaluation, especially for patients who cannot obtain sophisticated functional platelet testing, such as babies, those with severe thrombocytopenia, and those geographically distant from a specialized hematology or hemostasis laboratory. VUS remains highly prevalent, and novel variants are frequently discovered with varying predicted impact. Analyses such as these provide an avenue for research studies that will push our understanding of thrombopoiesis and platelet function forward.
