# Supplementary material for: Molecular diagnosis of inherited platelet disorder via a targeted whole-exome virtual gene panel: a 5-year institutional experience
Source: Res Pract Thromb Haemost. 2026 Mar 12;10(2):103413. doi: 10.1016/j.rpth.2026.103413 (PMC13092180; doi:10.1016/j.rpth.2026.103413)
Supplement: Supplementary Tables 1 and 2 [file mmc1.docx]

Supplementary table 1. Gene content of the 67-gene platelet disorder sequencing panel.

| **Gene** | **Inheritance** | **Condition** | **Effect on platelet** |
| --- | --- | --- | --- |
| *ABCG5* | AR | Macrothrombocytopenia and sitosterolemia | production |
| *ABCG8* | AR | Macrothrombocytopenia and sitosterolemia | production |
| *ACBD5* | AD | Autosomal dominant thrombocytopenias (PMID: 20626622) | production |
| *ACTN1* | AD | Bleeding disorder, platelet-type, 15 | production |
| *ANKRD26* | AD | Thrombocytopenia 2 | production |
| *ANO6 (TMEM16F)* | AR | Scott syndrome | function |
| *AP3B1* | AR | Hermansky-Pudlak syndrome 2 | function |
| *AP3D1* | AR | Hermansky-Pudlak syndrome 10 | function |
| *ARPC1B* | AR | Platelet abnormalities with eosinophilia and immune-mediated inflammatory disease | production |
| *BLOC1S3* | AR | Hermansky-Pudlak syndrome 8 | function |
| *BLOC1S6* | AR | Hermansky-Pudlak syndrome 9 | function |
| *CD36* | AR | Platelet glycoprotein IV deficiency | function |
| *CYCS* | AD | Thrombocytopenia 4 | production |
| *DIAPH1* | AD | Macrothrombocytopenia and hearing loss | production |
| *DTNBP1* | AR | Hermansky-Pudlak syndrome 7 | function |
| *ETV6* | AD | Thrombocytopenia 5 | production |
| *FERMT3* | AR | Leukocyte adhesion deficiency, type III | function |
| *FLI1* | AD/AR | Bleeding disorder, platelet-type, 21 | production |
| *FLNA* | X linked | Thrombocytopenia with periventricular nodular heterotopia (PVNH) | production |
| *FYB1* | AR | Thrombocytopenia 3 | production |
| *GATA1* | X linked | X-linked thrombocytopenia | production and function |
| *GFI1B* | AD/AR | Bleeding disorder, platelet-type, 17 | production and function |
| *GP1BA* | AD/AR; AD | Bernard-Soulier syndrome,type A2; Platelet-type von Willebrand disease | production and function |
| *GP1BB* | AR; AD | Bernard-Soulier syndrome; Mild macrothrombocytopenia | production and function |
| *GP6* | AR | Bleeding disorder, platelet-type, 11 | function |
| *GP9* | AR | Bernard-Soulier syndrome, type C | production and function |
| *HOXA11* | AD | Amegakaryocytic thrombocytopenia radio-ulnar synostosis | production |
| *HPS1* | AR | Hermansky-Pudlak syndrome 1 | function |
| *HPS3* | AR | Hermansky-Pudlak syndrome 3 | function |
| *HPS4* | AR | Hermansky-Pudlak syndrome 4 | function |
| *HPS5* | AR | Hermansky-Pudlak syndrome 5 | function |
| *HPS6* | AR | Hermansky-Pudlak syndrome 6 | function |
| *ITGA2* | AD | Glycoprotein Ia deficiency | function |
| *ITGA2B* | AR | Glanzmann’s thrombasthenia 1 | function |
| *ITGA2B* | AD; AD | Bleeding disorder platelet type 16 (AD); Macrothrombocytopenia (AD) | production |
| *ITGB3* | AR | Glanzmann’s thrombasthenia | function |
| *ITGB3* | AD | Bleeding disorder | production |
| *LYST (CHS1)* | AR | Chediak-Higashi syndrome | function |
| *MASTL* | AD | Autosomal dominant thrombocytopenias | production |
| *MECOM* | AD | Radioulnar synostosis with amegakaryocytic thrombocytopenia 2 | production |
| *MPIG6B* | AR | Thrombocytopenia, anemia, and myelofibrosis | production |
| *MPL* | AR | Congenital amegakaryocytic thrombocytopenia | production |
| *MYH9* | AD | Macrothrombocytopenia and Granulocyte Inclusions with or without Nephritis or Sensorineural Hearing Loss | production and function |
| *NBEA* | AD | Autism and dense granule deficiency | function |
| *NBEAL2* | AR | Gray platelet syndrome | function |
| *ORAI1* | AD | Stormorken syndrome | function |
| *P2RX1* | n/a | ADP receptor defects (PMID: 10816552) | function |
| *P2RY1* | possible AR | Moderate platelet-related bleeding phenotype with diminished platelet responsiveness to thrombin and thrombin-mimetic peptides in vitro. (PMID: 15514209, 21792575) | function |
| *P2RY12* | AR | Bleeding disorder, platelet-type, 8 | function |
| *PLA2G4A* | AR | Gastrointestinal ulceration, recurrent, with dysfunctional platelets | function |
| *PRKACG* | AR | Bleeding disorder, platelet-type, 19 | production and function |
| *PTGS1* | AD/AR | Platelet-type bleeding disorder 12 | function |
| *RAB27A* | AR | Griscelli syndrome, type 2 | function |
| *RASGRP2* | AR | Bleeding disorder, platelet-type, 18 | function |
| *RBM8A* | AR | Thrombocytopenia absent radius (TAR) syndrome | production |
| *RUNX1* | AD; AD/somatic | Familial platelet disorder with associated myeloid malignancy; Leukemia/MDS | production |
| *SLFN14* | AD | Bleeding disorder, platelet-type, 20 | production |
| *STIM1* | AD; AR | Stormorken syndrome; Immunodeficiency 10 | production |
| *STX11* | AR | Familial HLH type 4 | function |
| *STXBP2* | AR | Familial HLH type 5 | function |
| *TBXA2R* | AD | Bleeding disorder, platelet-type, 13, susceptibility to | function |
| *TBXAS1* | AR | Ghosal hematodiaphyseal syndrome | function |
| *THPO* | AD; AR | Thrombocytopenia 9; Amegakaryocytic thrombocytopenia, congenital, 2 | production |
| *TUBB1* | AD | Macrothrombocytopenia | production |
| *UNC13D* | AR | Familial HLH type 3 | function |
| *VIPAS39* | AR | Arthrogryposis-renal dysfunction-cholestasis syndrome 2 | function |
| *VPS33B* | AR | Arthrogryposis-renal dysfunction-cholestasis syndrome 1 | function |
| *VPS45* | AR | Congenital neutropenia & platelet α granule defect | function |
| *WAS* | X-linked | Wiskott-Aldrich syndrome/Thrombocytopenia | production |

AD, autosomal dominant; AR, autosomal recessive

Supplementary table 2. Follow up analysis may further clarify the variant significance for these 28 cases with inconclusive molecular diagnosis

| Pt ID | Age, Sex | Clinical Background | Plt (K/uL) | Prior Workup | Gene | Variant | Associated Disease | Variant Classification | Previous reported? | ClinVar; gnomAD;  In silico predictions | Diagnostic? |
| --- | --- | --- | --- | --- | --- | --- | --- | --- | --- | --- | --- |
| 83 | 9y, F | abnormal bleeding and platelet dysfunction | NA | NA | *ABCG5* | **NM_022436.2:c.1204_1208del p.(Phe402ArgfsTer92), het** | Sitosterolemia 2, (AR) | LP | This study | ClinVar: NA;  gnomAD: NA;  Prediction: loss of function | Uncertain (2^nd^ variant not detected) |
| 72 | 11y, M | platelet dysfunction, abnormal bleeding, and prolonged epistaxis | NA | NA | *ABCG8* | NM_022437.2:c.1476T>A p.(Tyr492Ter), het | Sitosterolemia 1, (AR) | Path | [24657386](http://www.ncbi.nlm.nih.gov/entrez/query.fcgi?cmd=Retrieve&db=PubMed&list_uids=24657386&dopt=Abstract), [36555767](http://www.ncbi.nlm.nih.gov/sites/entrez?cmd=Retrieve&db=PubMed&list_uids=36555767&dopt=Abstract) | ClinVar: 1441157 (Path);  gnomAD-all: 0.0036%;  gnomAD-max (Admixed American): 0.0087%;  Prediction: loss of function | Uncertain (2^nd^ variant not detected) |
| 68 | 8y, F | NA | NA | NA | *ACTN1* | NM_001130004.1:c.1019C>T p.(Thr340Met), het | Bleeding Disorder, Platelet-Type, 15, (AD) | VUS | [32581362](http://www.ncbi.nlm.nih.gov/entrez/query.fcgi?cmd=Retrieve&db=PubMed&list_uids=32581362&dopt=Abstract), [35295078](http://www.ncbi.nlm.nih.gov/sites/entrez?cmd=Retrieve&db=PubMed&list_uids=35295078&dopt=Abstract) | ClinVar: 812968(VUS);  gnomAD: not reported;  In silico predictions: deleterious | Uncertain |
| 79 | 6y, F | unexplained thrombocytopenia | NA | NA | *ACTN1* | **NM_001130004.1:c.2200C>A p.(Gln734Lys), het** | Bleeding Disorder, Platelet-Type, 15, (AD) | VUS | This study | ClinVar: 2579802(VUS);  gnomAD: NA;  In silico predictions: deleterious | Uncertain |
| 11 | 16y, M | thrombocytopenia, leukopenia, family history of bleeding | NA | NA | *ANKRD26* | **NM_014915.2:c.1444A>G (p.Met482Val) het** | Thrombocytopenia 2, (AD) | VUS | This study | ClinVar: 2573893 (VUS);  gnomAD-all: 0.0036%;  gnomAD-max (European-non Finnish): 0.0080%;  In silico predictions: no impact | Uncertain |
| 18 | 38y, F | easy bruising, menorrhagia | 226 | Normal CBC, PTT, thrombin time, von Willebrand studies, factor VIII | *ANO6* | **NM_001025356.2:c.1903C>T p.(Arg635*), het** | Scott Syndrome, (AR) | Path | This study | ClinVar: NA;  gnomAD: NA;  In silico predictions: loss of function | Uncertain (2^nd^ variant not detected) |
| 17 | 20y, F | recurrent epistaxis, heavy menses | 266 | Normal CBC, PT, PTT, fibrinogen, von Willebrand studies, and platelet function tests | *ANO6* | **NM_001204803.1:c.2055_2056del p.(Phe685Leufs*2), het** | Scott Syndrome, (AR) | Path | This study | ClinVar: NA;  gnomAD-all: 0.0025%;  gnomAD-max (European-non Finnish): 0.0054%;  Prediction: loss of function | Uncertain (2^nd^ variant not detected) |
| 78 | 18y, M | unexplained thrombocytopenia | NA | NA | *FLNA* | NM_001456.4:c.6752T>C p.(Phe2251Ser), hemi | Thrombocytopenia with periventricular nodular heterotopia, (XL) | VUS | [31064749](http://www.ncbi.nlm.nih.gov/entrez/query.fcgi?cmd=Retrieve&db=PubMed&list_uids=31064749&dopt=Abstract) | ClinVar: 577159 (Uncertain)  gnomAD-all: 0.0006%  gnomAD-max (European (Non-Finnish)): 0.0012%  In silico predictions: deleterious | Uncertain |
| 80 | 38y, F | macrothrombocytopenia | NA | NA | *FYB1* | **NM_001243093.1:c.53dup p.(Asn19GlnfsTer10), het** | Thrombocytopenia 3, (AR) | LP | This study | ClinVar: NA;  gnomAD-all: 0.0004%  gnomAD-max (European (Non-Finnish)): 0.0009%  Prediction: loss of function | Uncertain (2^nd^ variant not detected) |
| 73 | 5m, M | a history of unexplained thrombocytopenia | NA | NA | *GATA1* | **NM_002049.3:c.1046T>C p.(Val349Ala), hemi** | Thrombocytopenia, X-linked, with or without dyserythropoietic anemia, (XLR); Thrombocytopenia with beta-thalassemia, X-linked, (XLR);  Leukemia, megakaryoblastic, with or without Down syndrome, somatic | VUS | This study | ClinVar: 1047760 (VUS);  gnomAD-all: 0.0022%, 0 hemi;  gnomAD-max (European-non Finnish): 0.0229%;  In silico predictions: no impact | Uncertain |
| 77 | 15y, M | unexplained thrombocytopenia | NA | NA | *GATA1* | **NM_002049.3:c.626C>G p.(Ala209Gly), hemi** | Thrombocytopenia, X-linked, with or without dyserythropoietic anemia, (XLR); Thrombocytopenia with beta-thalassemia, X-linked, (XLR);  Leukemia, megakaryoblastic, with or without Down syndrome, somatic | VUS | This study | ClinVar: NA;  gnomAD: NA;  In silico predictions: deleterious | Uncertain |
| 85 | 2y, F | unexplained thrombocytopenia; father and brother affected | 88-97 | CBC: elevated MPV (12.8-13.8fl) | *GP1BA* | NM_000173.6:c.449A>G p.(Asn150Ser), het | Bernard-Soulier Syndrome, Type A2, (AD) | VUS (for AD BSS) | [29082515](http://www.ncbi.nlm.nih.gov/entrez/query.fcgi?cmd=Retrieve&db=PubMed&list_uids=29082515&dopt=Abstract), [31064749](http://www.ncbi.nlm.nih.gov/sites/entrez?cmd=Retrieve&db=PubMed&list_uids=31064749&dopt=Abstract) | ClinVar: 627076(LP);  gnomAD: NA;  In silico predictions: deleterious | Uncertain |
| 20 | 15y, M | Thrombocytopenia, petechiae, mom and brother affected | NA | von Willebrand testing normal | *GP1BA* | NM_000173.6:c.1845_1849del p.(Asn616Profs*29), het | Bernard-Soulier Syndrome, type A1, (AR) | Path (for AR BSS) | [34662886](http://www.ncbi.nlm.nih.gov/entrez/query.fcgi?cmd=Retrieve&db=PubMed&list_uids=34662886&dopt=Abstract) | ClinVar: NA;  gnomAD-all: 0.0446%;  gnomAD-max (European-Finnish): 0.1000%;  Prediction: loss of function | Uncertain (2^nd^ variant not detected) |
| 76 | 26y, F | mild thrombocytopenia of unclear etiology | 94-119 | Elevated MPV (15.5-15.9 fL); normal platelet EM and platelet glycoprotein expression | *GP1BB* | NM_000407.4:c.137G>A p.(Trp46Ter), het | Bernard-Soulier syndrome, type B, (AR) Giant platelet disorder, isolated, (AR) | Path | [10887115](http://www.ncbi.nlm.nih.gov/entrez/query.fcgi?cmd=Retrieve&db=PubMed&list_uids=10887115&dopt=Abstract), [32581362](http://www.ncbi.nlm.nih.gov/sites/entrez?cmd=Retrieve&db=PubMed&list_uids=32581362&dopt=Abstract) | ClinVar: 16040(LP);  gnomAD: NA;  In silico predictions: loss of function | Uncertain (2^nd^ variant not detected) |
| 74 | 62y, M | longstanding thrombocytopenia without significant bleeding; brother and a paternal cousin affected. | 70-110 | No macrothrombocytes on blood smear | *GP9*  *ANKRD26* | NM_000174.4:c.488C>A p.(Ala163Asp), homo  NM_014915.2:c.3058C>T p.(Arg1020Cys), het | Bernard-Soulier syndrome, type C, (AR)  Thrombocytopenia 2,  (AD) | VUS  VUS | [*26226975*](http://www.ncbi.nlm.nih.gov/entrez/query.fcgi?cmd=Retrieve&db=PubMed&list_uids=26226975&dopt=Abstract) | ClinVar: 639283(VUS);  gnomAD-all: 0.0090%, 0 hemi;  gnomAD-max (African American): 0.0955%;  In silico predictions: conflicting  ClinVar: 2884260(VUS);  gnomAD-all: 0.0021%, 0 hemi;  gnomAD-max (Ashkenazi Jewish 0.0193%;  In silico predictions: conflicting | Uncertain  Uncertain |
| 86 | 14y, M | NA | NA | NA | *GP9* | **NM_000174.4:c.461T>C p.(Leu154Pro), homo** | Bernard-Soulier syndrome, type C, (AR) | VUS | *This study* | ClinVar: 1337399(VUS);  gnomAD-all: 0.0097%, 0 hemi;  gnomAD-max (African American): 0.0546%;  In silico predictions: conflicting | Uncertain |
| 69 | 9y, F | with unspecified symptoms | NA | NA | *HPS4* | **NM_022081.5:c.670-7_691del p.(?), het** | Hermansky-Pudlak syndrome 4, (AR) | LP | This study | ClinVar: NA;  gnomAD: NA;  Prediction: 29 bp deletion predicted to disrupt the splice acceptor of Intron 8 | Uncertain (2^nd^ variant not detected) |
| 81* | 13y, M | plt dysfunction/bleeding symptoms | NA | NA | *ITGA2B* | **NM_000419.4:c.2095-15A>G p.(?), homo** | Glanzmann Thrombasthenia 1, (AR) | VUS | This study | ClinVar: NA;  gnomAD: NA;  In silico predictions: uncertain splicing effect | Uncertain |
| 82* | 6y, F | plt dysfunction | NA | NA | *ITGA2B* | **NM_000419.4:c.2095-15A>G p.(?), homo** | Glanzmann Thrombasthenia 1, (AR) | VUS | This study | ClinVar: NA;  gnomAD: NA;  In silico predictions: uncertain splicing effect | Uncertain |
| 66 | 16y, M | NA | NA | NA | *ITGB3* | **NM_000212.2:c.778-2A>G p.(?), het** | Glanzmann Thrombasthenia 2, (AR) | LP (for GT) | This study | ClinVar: 623143(VUS);  gnomAD-all: 0.0008%;  gnomAD-max (East Asian): 0.0109%;  In silico predictions: predicted to disrupt the donor acceptor | Uncertain (2^nd^ variant not detected) |
| 67 | 35y, F | unexplained mild thrombocytopenia that worsened during her pregnancy | NA | Blood smear is notable for large platelets | *ITGB3* | NM_000212.2:c.655G>A p.(Val219Met), het | Glanzmann Thrombasthenia 2, (AR) | LP (for GT) | [11722423](http://www.ncbi.nlm.nih.gov/entrez/query.fcgi?cmd=Retrieve&db=PubMed&list_uids=11722423&dopt=Abstract), [37647632](http://www.ncbi.nlm.nih.gov/sites/entrez?cmd=Retrieve&db=PubMed&list_uids=37647632&dopt=Abstract) | ClinVar: 2498348(LP);  gnomAD-all: 0.0012%;  gnomAD-max (South Asian): 0.0033%;  In silico predictions: deleterious | Uncertain (2^nd^ variant not detected) |
| 70 | 18y, F | patient with low VWF and mild thrombocytopenia whose bleeding symptoms (petechiae and bruising, and history of iron deficiency) appear disproportionate to laboratory findings. Positive family history of bleeding | NA | Mild thrombocytopenia with increased MPV, low VWF (40's) RCoF activity and proportional VWF antigen, normal whole blood platelet aggregation x2 | *MASTL* | **NM_001172303.2:c.1935del p.(Leu646Ter), het** | Thrombocytopenia (AD) | VUS | This study | ClinVar: NA;  gnomAD: NA;  In silico predictions: loss of function | Uncertain |
| 12 | 17y, F | easy bleeding and bruising, thrombocytopenia; dad with AML | NA | Abnormal platelet function testing | *RUNX1* | **NM_001754.4:c.939_950del (p.Ala315_Ser318del), likely het. (VAF=0.40)** | Platelet disorder, familial, with associated myeloid malignancy, (AD); Leukemia, acute myeloid, (AD, somatic) | VUS | This study | ClinVar: 532672(LB);  gnomAD-all: 0.0036%;  gnomAD-max (European-non Finnish): 0.0080%;  Prediction: in frame 4-amino acid deletion in the transcription factor, Runt-related, RUNX domain | Uncertain |
| 19 | 17y, M | easy bruising and bleeding, post-operative hemorrhage | 196 | Normal CBC, PT, PTT, von Willebrand profile, platelet function testing abnormal in response to ADP | *STXBP2* | NM_006949.3:c.1247-1G>C p.(?), het | Familial HLH (AR);  Platelet granule release deficits (AD) | Path | 20823128, 23687090, 19804848 | ClinVar: 330555 (Path);  gnomAD-all: 0.0020%;  gnomAD-max (European-non Finnish): 0.0037%;  In silico predictions: predicted to disrupt splicing | Uncertain (2^nd^ variant not detected) |
| 16 | 4m, M | 22q11.2 deletion syndrome, arterial thrombus, mild thrombocytopenia | 118 | Serial CBC, PT, PTT, fibrinogen | *TBXAS1*  *STXBP2* | **NM_001061.4:c.961_965dup p.(Met323Alafs*7), het**  NM_006949.3:c.1254_1257del p.(Ser418Argfs*7), het | Ghosal hematodiaphyseal syndrome (AR)  Familial HLH (AR)  Platelet granule release deficits (AD) | LP  Path | This study  21152410 | ClinVar: NA;  gnomAD-all: 0.0014%;  gnomAD-max (East Asian): 0.0201%;  In silico predictions: loss of function  ClinVar: NA;  gnomAD: NA;  Predictions: loss of function | Uncertain (2^nd^ variant not detected)  Uncertain (2^nd^ variant not detected) |
| 75 | 1y, M | excessive bleeding and bruising and moderate thrombocytopenia with normal MPV | NA | normal MPV | *THPO* | **NM_000460.3:c.296G>A p.(Arg99Gln), het, maternally inherited** | Thrombocytopenia (AD, AR) | VUS | This study | ClinVar: NA;  gnomAD-all: 0.0032%;  gnomAD-max (Admixed American): 0.0056%;  In silico predictions: conflicting | Uncertain(2^nd^ variant not detected) |
| 65 | 21y, F | longstanding thrombocytopenia without bleeding history. Her father (plt 90-110K/uL) and 2 other family members are also affected. A brother with thrombocytopenia carries the same variants. | 100-115 | MPV:13.5-14.4fL. | *TUBB1* | **NM_030773.3:c.1033A>T p.(Ile345Phe), het**  **NM_030773.3:c.173A>G p.(Lys58Arg), het** | TUBB1-Related Macrothrombocytopenia (AD) | VUS  VUS | This study  This study | ClinVar: NA;  gnomAD: NA;  In silico predictions: deleterious  ClinVar: NA;  gnomAD: NA;  In silico predictions: conflicting | Uncertain (phase unknown) |
| 71 | 20y, F | history of mild to moderate thrombocytopenia with a mild bleeding tendency | NA | Normal VWF with elevated factor VIII and normal aPTT | *TUBB1* | **NM_030773.3:c.1257_1260delinsTGAGTACCATGTT p.(Ser420delinsGluTyrHisVal), het** | TUBB1-Related Macrothrombocytopenia (AD) | VUS | This study | ClinVar: NA;  gnomAD: NA;  Prediction: inframe indel in the last exon | Uncertain |

*81 and 82 are siblings. Previously unreported variants are indicated in bold.

AD, autosomal dominant; AR, autosomal recessive; XL, X-linked; XLR, X-linked recessive; Path, pathogenic; LP, likely pathogenic; VUS, variant of unknown significance; het, heterozygous; hemi, hemizygous; homo, homozygous; VAF, variant allele fraction; HI, haploinsufficiency; gnomAD-genome aggregation database v.2.1.0.

ADP, adenosine diphosphate; AML, acute myeloid leukemia; BM, bone marrow; CBC, complete blood count; EM, electron microscopy; fl, fluid ounce; F, female; M, male; m, months old; y, years old; MCV, mean corpuscular volume; MDS, myelodysplastic syndrome; MPV, mean platelet volume; NA, not available; Plt, platelet count; Pt, patient; PT, prothrombin time; PTT, partial thromboplastin time; aPTT, activated partial thromboplastin time; RCoF, Ristocertin Cofactor; VWF, von willebrand factor.
